# Lyophilized Chitosan-Based Hydrogels as a Potential Stimuli-Responsive Carrier System for Anti-Inflammatory Drugs: Ibuprofen Solubility Modulation at Variable pH of Simulated GIT Conditions

**DOI:** 10.3390/polym18121537

**Published:** 2026-06-20

**Authors:** Veronika Mikušová, Jarmila Prieložná, Dominika Žigrayová, Michal Hanko, Peter Mikuš

**Affiliations:** 1 Department of Galenic Pharmacy, Faculty of Pharmacy, Comenius University Bratislava, Odbojarov 10, SK-832 32 Bratislava, Slovakia; mikusova@fpharm.uniba.sk (V.M.); jarmila.ferkova@uniba.sk (J.P.); zigrayova1@uniba.sk (D.Ž.); 2 Department of Pharmaceutical Analysis and Nuclear Pharmacy, Faculty of Pharmacy, Comenius University Bratislava, Odbojarov 10, SK-832 32 Bratislava, Slovakia; hanko11@uniba.sk; 3 Toxicological and Antidoping Center, Faculty of Pharmacy, Comenius University Bratislava, Odbojarov 10, SK-832 32 Bratislava, Slovakia

**Keywords:** chitosan hydrogel lyophilizates, ibuprofen, solubility enhancement, dissolution profiles, drug release, GIT simulating model

## Abstract

Poor aqueous solubility and consequently low bioavailability of various NSAIDs (non-steroidal anti-inflammatory drugs) usually result in high and multiple dosing with potentially serious side effects. Therefore, systems for the effective transport of NSAIDs through the GIT (gastrointestinal tract), ensuring enhanced bioavailability, remain in high demand. In the present work, we studied chitosan (CS) hydrogel lyophilizates as carrier systems for a model NSAID, namely ibuprofen (IBU). The CS-IBU lyophilizates were prepared from homogeneous or heterogeneous CS-IBU hydrogels to assess their influence on the resulting lyophilizate microstructure and IBU dissolution profiles. To gain a complex view of the CS-IBU behavior and its practical consequences, dissolution profiles of free IBU (reference) and CS-associated IBU (CS-IBU) were examined and compared to each other at variable pH (1.2 and 6.5) in two separate dissolution systems and in one discontinuous dissolution system mimicking GIT conditions. The results of dissolution experiments were supported by kinetic model data. This study demonstrated that the dissolution of IBU from the CS-IBU lyophilizates is affected by two main pH-dependent competitive effects; i.e., dissolved CS acts as an IBU solubilizer and the undissolved CS matrix serves as an IBU trap, which could be used in the rational design of innovative stimuli (pH)-responsive oral dosage forms of IBU.

## 1. Introduction

Drug delivery systems (DDS) are pharmaceutical formulations used to transport therapeutic drugs and other bioactive molecules into the body as needed, safely achieving the desired therapeutic effect. Novel DDS are rationally designed to enhance the delivery and performance of existing drugs compared with traditional systems. Novel DDS combine advanced techniques and new dosage forms to target, control, and modulate the delivery of drugs. Such systems are usually designed to improve the aqueous solubility and chemical stability of active agents, increase their efficacy, safety, and patient compliance, and reduce side effects [1,2].

Drug delivery systems based on biodegradable polymers may be particularly advantageous over other carriers for the controlled release of drugs because of their improved therapeutic efficacy for a variety of low-molecular-weight drugs, reduced toxicity, controlled diffusion, and targeted delivery [3].

One of the polymers that receives significant interest for such applications is chitosan (CS). CS is a natural biopolymer composed of β-[1 → 4]-linked 2-acetamido-2-deoxy-d-glucopyranose and 2-amino-2–2-deoxy-d-glucopyranose units and may have varying degrees of deacetylation and molecular weight [4,5]. CS can be physically conditioned into films, gel beads, or fibers owing to its solubility in mildly acidic media. CS has been shown to exhibit adhesive, wound-healing, and antimicrobial properties [3]. However, its application as a matrix to release drugs is limited due to its low solubility in water under physiological conditions and lack of amphipathicity. Meanwhile, several CS derivatives obtained from the exploitation of a series of reactions resulted in improvement in these biopolymers and their applicability [6]. CS and its derivatives have been reported in several recent works demonstrating the potentialities of these polysaccharides for the delivery of NSAIDs [7,8,9,10,11,12].

Ibuprofen (IBU) [(RS)-2-(4-(2-methylpropyl)phenyl)propanoic acid] was chosen as the model drug in this study because it is an effective NSAID used for the management of pain, fever, and symptoms of rheumatoid arthritis and osteoarthritis. It is a high-dose non-selective reversible inhibitor of cyclooxygenase enzymes (COX-1 and COX-2), which are responsible for the production of prostaglandins (PGE 2 and PGI 2) from arachidonic acid [13]. However, because IBU is practically insoluble in water (0.021 mg·mL^−1^) [14], classified in class 2 of the Biopharmaceutical Classification System (BCS) (low solubility at pH 1.2 and 4.5 and high solubility at pH 6.8) [15], and has a short biological half-life (2 h) and rapid clearance after oral administration, high and multiple dosing are usually required, which in turn may lead to wasted dosing and potentially serious side effects such as ulceration and bleeding [16]. It is worth mentioning that there is an inconvenience in the use of non-steroidal anti-inflammatory drugs, often associated with gastrointestinal complications. A total of 15–30% of patients who utilize these kinds of drugs for a long time have gastrointestinal ulcers and bleeding or renal dysfunction. Thus, the need arises to minimize these adverse effects and to extend its inflammatory action, where the delivery of the drug using a suitable matrix, such as a hydrogel based on CS or CS derivatives, can help to reduce a series of undesirable side effects [17].

One of the approaches for the formulation of IBU is its integration into the CS hydrogel structure, as demonstrated by several recent papers [18,19,20,21]. Several studies showcased that ibuprofen associates with chitosan predominantly through ionic pairing between protonated amines on chitosan and the carboxylate of ibuprofen, and to a lesser extent, through hydrogen bonding and hydrophobic interactions. FT−IR analysis of low-molecular-weight chitosan–ibuprofen complexes showed characteristic shifts in the ibuprofen carboxylate bands consistent with conversion from sodium–carboxylate to ammonium–carboxylate ion pairs. An additional 1H NMR analysis revealed upfield shifts in hydrogen signals of chitosan upon complexation, and the results of differential scanning calorimetry further confirmed the existence of ionic interactions based on the disappearance of the endothermic peak of ibuprofen [22]. Consistent results were obtained from the study of chitosan–ibuprofen aerogels, where FT−IR analysis showed negative band shifts in amine and hydroxyl groups of chitosan, indicating strong intermolecular hydrogen bonding. Altered X-ray diffraction, differential calorimetric, and thermogravimetric profiles further indicated association and hydrogen bonding [23]. pH-dependent drug release studies using chitosan and its chemical derivatives further demonstrated that chemically modified chitosan bearing additional hydrogen bond donors/acceptors provides additional stabilization of the chitosan–ibuprofen complex. Based on the results obtained, it was concluded that the chitosan–ibuprofen interaction is through an ion-pairing mechanism with a contribution of hydrogen bonding and hydrophobic effects [17].

There were several approaches demonstrated in the literature for the formulation of IBU into CS hydrogel. The most used IBU solution (usually ethanolic) is added to the CS hydrogel to form a dispersion [24] because of the acidic pH of the CS solution. In this case, the drug release can be affected by the particle size [25] of suspended IBU, and it is difficult to maintain the small polydispersion index reproducibility. To avoid this problem, various approaches were studied. One of the possibilities is to use a derivative of CS, which is soluble in ethanol, for example, carboxymethyl–hexanoyl chitosan [26]. However, when using native CS, some authors tried to incorporate IBU into the hydrogel dissolved in an oil phase. For example, Dejkic et al. [18] dissolved IBU in the oil phase and incorporated IBU-loaded microemulsion into CS hydrogel. Mahmood et al. [27] prepared IBU-loaded CS–lipid nanoconjugate hydrogel using Phospholipon 906. Other authors tried to use solubilizers. Morgado et al. [28] prepared β-cyclodextrins complexes with IBU at supercritical conditions to incorporate IBU in CS/PVA dressings intended for wound healing. A different approach is to prepare nanoparticles loaded with IBU and incorporate them into a chitosan hydrogel. In recent papers, nanoparticles (NPs) such as mesoporous silica NPs [29], solid-lipid NPs [30], ZnO nanocomposite beads [31], silver NPs [32], CuO NPs [33], or graphene oxide NPs [34] were studied for IBU loading, and they were incorporated in CS gel. In the case of CS hydrogel being a material for the preparation of films or membranes, CS membranes can be immersed in IBU solution, and the drug is allowed to permeate into the membrane [35]. It is clear from the above overview that there is still interest in the investigation of new procedures for the effective incorporation of IBU into CS hydrogel and related forms (CS nanoparticles, CS films, etc.), as a prerequisite step in developing new IBU delivery systems. One option for incorporating IBU into CS could be a lyophilized CS hydrogel derived from either a heterogeneous (disperse) or homogeneous hydrogel precursor. Lyophilized hydrogels for oral administration represent an important advancement because they combine the controlled-release properties of hydrogels with the stability advantages of freeze-drying. Their highly porous structure enables rapid rehydration and can improve the dissolution and bioavailability of poorly soluble drugs compared with conventional tablets or capsules [36]. Lyophilization also enhances the stability and shelf life of moisture-sensitive drugs by reducing hydrolytic degradation. Unlike immediate-release dosage forms, these systems can provide sustained and site-specific drug release within the gastrointestinal tract. Their mucoadhesive and swelling properties may prolong gastrointestinal residence time and improve drug absorption. Overall, lyophilized oral hydrogels offer improved stability, controlled release, and enhanced therapeutic efficiency compared with many traditional oral drug delivery systems [37].

To the best of our knowledge, a comprehensive evaluation of hydrogel lyophilizate, as a CS-IBU formulation for IBU solubility control, has not yet been presented so far.

Therefore, this work aims to prepare lyophilized CS-IBU hydrogels from their homogeneous and heterogeneous hydrogel precursors and study their IBU dissolution properties under different acid-base conditions mimicking the GIT to assess their practical potentialities as IBU stimuli (pH)-responsive carriers. To comprehensively evaluate the impact of different parameters (CS/IBU ratio, lyophilizate microstructure resulting from different hydrogel preparations, and pH of the dissolution media) on IBU dissolution profiles, the dissolution experiment was correlated with calculated kinetic models, SEM microstructure analysis, and swelling index measurements. Possibilities for oral administration of the lyophilized CS-IBU dose and its potential behavior in the GIT are discussed with respect to the obtained experimental results carried out in simulated continuous and discontinuous GIT systems.

## 2. Materials and Methods

### 2.1. Chemicals

Chemicals were purchased: chitosan MMW (Sigma Aldrich, St. Louis, MO, USA), lactic acid 80% (Centralchem s.r.o., Bratislava, Slovakia), acetic acid (SLAVUS s.r.o., Bratislava, Slovakia), isopropyl alcohol (Centralchem s.r.o., Bratislava, Slovakia), ethanol 96% (Fagron a.s., Olomouc, Czech Republic), hydrochloric acid (Centralchem s.r.o., Bratislava, Slovakia), sodium hydroxide (MIKROCHEM spol. s.r.o., Pezinok, Slovakia), deionized water (Biosan, Ljubljana-Crnuce, Slovenia), and ibuprofen Ph. Eur. (Fagron a.s., Olomouc, Czech Republic).

### 2.2. Instrumentation

UV-VIS Spectrophotometer: UV-1900i (Shimadzu Corporation, Kyoto, Japan); freeze-dryer (lyophilizer): Alpha 1-2 LDplus (Martin Christ, Osterode am Harz, Germany); dissolution paddle apparatus: AT7 (SOTAX, Aesch, Switzerland); scanning electron microscope: SNE-4500M Plus (SEC Co., Ltd., Suwon-si, Republic of Korea); ion sputter coater: MCM-100P (SEC Co., Ltd., Suwon-si, Republic of Korea); FT−IR spectrometer: Spectrum Two (PerkinElmer, Springfield, IL, USA); analytical balance: Radwag PS 600/C2 (Radwag, Radom, Poland); pH meter: FiveGo F2 (Mettler Toledo, Greifensee, Switzerland); and water purification system: Labaqua BIO (Biosan, Riga, Latvia).

### 2.3. Preparation of CS Hydrogels

CS-based hydrogels (100.0 g) were prepared by dispersing medium-molecular-weight chitosan (CS MMW; 0.2, 0.4, or 0.8 g) in deionized water acidified with (i) 2.0 g of acetic acid (reference/comparative CS hydrogel system), (ii) 1.3 mL of 80% (*w*/*w*) lactic acid and 45.0 g of 96% (*v*/*v*) ethanol, or (iii) 1.3 mL of 80% (*w*/*w*) lactic acid and 40.0 g of isopropyl alcohol. The final mass of each formulation was then adjusted to 100.0 g with deionized water. The dispersions were magnetically stirred at laboratory temperature (22–25 °C) at 500 rpm for 2 h. After mixing, the formulations were stored in a refrigerator (4–8 °C) for at least 24 h to allow complete swelling of the CS network. The CS concentrations of 0.2%, 0.4%, and 0.8% (*w*/*w*) were selected based on preliminary experiments as a practical range providing physically stable and easily handleable hydrogels suitable for subsequent lyophilization and further characterization. Each formulation was prepared in three independent batches following the same protocol to confirm the reproducibility of the preparation process.

### 2.4. Preparation of CS Hydrogels Containing Ibuprofen

IBU-loaded hydrogels (100.0 g) were prepared using the CS-based formulations described above at selected CS concentrations. The target IBU content was 0.4% (*w*/*w*) in the final hydrogels. In the case of the hydrogels containing acetic acid, IBU was first suspended in 1.4 g of concentrated ethanol and subsequently added to the preformed CS hydrogel under magnetic stirring at laboratory temperature (22–25 °C) at approximately 500 rpm. Stirring continued for 2 h to ensure uniform dispersion of the drug within the polymer network. Owing to the limited solubility of IBU in this system, the resulting formulation was a suspension-type hydrogel (reference/comparative CS-IBU hydrogel system). For hydrogels containing lactic acid and ethanol or lactic acid and isopropyl alcohol, IBU was incorporated in an analogous way, after dissolution in a small volume of the respective alcohol, followed by addition to the chitosan hydrogel under magnetic stirring at approximately 500 rpm for 2 h at 22–25 °C until a homogeneous system was obtained (i.e., with dissolved both CS and IBU as well). After drug incorporation, all IBU-loaded hydrogels were stored in a refrigerator (4–8 °C) for at least 24 h to allow complete swelling and equilibration of the polymer network and drug distribution. IBU-loaded hydrogels remained in the acidic pH range, i.e., pH 3.1–3.6 for the HAc system and pH 3.4–3.9 for the lactic acid systems (with either IPA or EtOH cosolvent). No further pH adjustment was performed. Each formulation was prepared in three independent batches following the same protocol to confirm the reproducibility of the preparation process.

### 2.5. Preparation of Lyophilizates

All CS hydrogels (previously cooled in a refrigerator, as stated in Section 2.4) were poured into Petri dishes, with an accurately weighed amount of 10.0 g per dish. The samples were then subjected to freeze-drying in a laboratory freeze-dryer. In the used configuration, the freeze-dryer was equipped with non-heated shelves, and heat was transferred to the samples mainly by radiation and conduction from the interior of the drying chamber. Before drying, the loaded hydrogels were frozen in the chamber, and subsequently, the pressure was reduced to approximately 0.021 mbar. Under these conditions, primary drying (sublimation of ice) was carried out for 18 h, followed by a secondary drying (desorption of bound water) step for an additional 6 h, resulting in a total process time of 24 h. Throughout the drying cycle, the ice condenser temperature was maintained at approximately −55 °C, corresponding to a water vapor pressure sufficiently low to support sublimation during primary drying and effective removal of residual moisture during secondary drying. After completion of freeze-drying, the obtained lyophilizates were immediately transferred to a desiccator and stored under dry conditions to minimize moisture uptake until further analysis.

### 2.6. SEM Analysis and Porosity Analysis

SEM analysis was performed using a tabletop scanning electron microscope SNE-4500M Plus (SEC Co., Ltd., Suwon-si, Republic of Korea). An ion sputter coater was used to coat the samples before microscopic visualization.

Random portions of the lyophilized samples were cut from the bulk material and fixed on a metallic holder using double-sided carbon tape. The fixed samples were subsequently coated with an approximately 8 nm thick layer of gold using an ion sputter coater.

Microscopic images of the lyophilizates were acquired using a scanning electron microscope operating in secondary electron mode under high vacuum, with an accelerating voltage of 10 kV and a working distance ranging from 8 to 13 mm, as indicated in the information panels at the bottom of each image.

Porosity analysis was in SEM images of CS lyophilizates (3 images taken from different areas of the lyophilizate) with the aid of the software ImageJ (version 1.54k). Porosity was expressed as

(1) $$porosity\left(\%\right)=\frac{Area\;of\;pores}{Total\;image\;area}\times 100$$

### 2.7. FT−IR Analysis

The FT−IR (Spectrum Two, PerkinElmer, Springfield, IL, USA) analysis of CS, IBU, and lyophilized hydrogel samples was performed using the ATR technique in the mid-infrared range (4000–450 cm^−1^) by acquiring 10 scans for each sample. Prior to the analysis and between each spectral acquisition, the ATR diamond crystal was cleaned using a cotton bud moistened with isopropyl alcohol. The FT−IR spectra were recorded after the samples were placed on the diamond crystal and pressed with a pressure arm to ensure good contact with the crystal surface. The spectra were analyzed without additional smoothing or baseline correction. To better visualize the fingerprint region of the acquired spectra, the scale of the x-axis was changed from 500 cm^−1^ to 250 cm^−1^ within the 2000–450 cm^−1^ range.

### 2.8. Swelling Index

The swelling index (SI) was determined for each CS lyophilizate containing IBU in three parallel measurements. Lyophilizates with Petri’s dishes were weighed (mass W_1_), then incubated at 37 ± 1 °C for 30 min. The swelling index was determined in aqueous media adjusted to pH 1.2 and pH 6.5. Both media were prepared from 0.1 M hydrochloric acid solution, and the pH was adjusted to 1.2 or 6.5, respectively, by the addition of 1 M sodium hydroxide. Thus, the media were individually composed and were identical to the media used later in the in vitro dissolution experiments (for dissolution tests, this approach was favored due to easy pH change in a discontinuous system simulating GIT conditions). The pH of the media was adjusted and checked with a calibrated pH meter, with an accuracy of approximately ±0.05 pH units. In total, 15 mL of dissolution medium with pH 1.2 and 6.5, respectively, was then pipetted on the surface of each lyophilizate, and the lyophilizates were allowed to swell in an incubator at 37 ± 1 °C. After 5 min of incubation, the excess liquid on the surface was carefully poured out, and the outer walls of the Petri dish were dried with filter paper. The swelled lyophilizate was then weighed again (mass W_2_). The process was repeated at intervals of 5, 10, 20, and 30 min. The swelling index was calculated according to the equation

(2) $$SI=\left(\frac{W_{2}-W_{1}}{W_{1}}\right)\times 100$$

where w_1_ is the initial weight of the dry sample, and w_2_ is the weight of the swollen hydrogel after the addition of the swelling medium.

### 2.9. In Vitro Drug Release

The dissolution profile of IBU from CS lyophilized hydrogels was determined by using a dissolution paddle apparatus (Apparatus 1 according to Ph. Eur. 11). The glass vessels were filled with 500 mL of 0.1 M hydrochloric acid with a pH of 1.2. They were maintained at 37 ± 0.5 °C. In 5 vessels, parallel measurements of the sample were made, and the sixth compartment was designated for a blank (the blank contained the same hydrochloric acid solution as the other compartments, but CS lyophilizate was prepared without IBU). Then, 5.0 mL of the medium was withdrawn at intervals of 15, 30, 45, 60, 90, and 120 min. The same volume of dissolution medium was replaced for each sample. Samples were analyzed for IBU content spectrophotometrically at λmax = 264 nm. The experimental results were expressed as mean ± SD (*n* = 5). In the second experiment, the same protocol was repeated using a dissolution medium of pH 6.5 (every compartment was filled with 500 mL of 0.1 M HCl, pH-adjusted to the range of 6.3–6.5 with the aid of 1 M NaOH solution). This time, the sampling intervals were the same as in the first experiment, plus 150, 180, 210, and 240 min. The third experiment began at a pH of 1.2. After 120 min, the pH of the medium in each vessel was adjusted to 6.5 using 0.2 M NaOH over 5 min. The dissolution test then continued, and the sampling intervals were the same as in the second experiment.

The tests were repeated for pure IBU. Then, 0.4 g of IBU was dissolved in 2.0 mL of conc. Ethanol was poured into a Petri dish. Ethanol was left to evaporate for 24 h at laboratory temperature, and the same dissolution tests were performed as with CS lyophilizates.

Mean dissolution time (MDT) was calculated according to the equation

(3) $$MDT=\frac{\Sigma t_{mid}∆M}{∆M}$$

where t_mid_ is the mean time between two sampling points, and ∆M is the amount of drug released between two time points.

### 2.10. Drug Release Kinetics

Five kinetic models were investigated as potential models for IBU release from lyophilizates. The models and appropriate equations are shown in Table 1. To calculate the kinetic constants and the correlation coefficient, Microsoft Excel was used.

### 2.11. Arrangement of the Experiments

Studied CS-IBU lyophilizates are intended to represent model solid drug-carrier systems for oral administration. For this purpose, we tested IBU dissolution in different acid-base environments (pH 1.2, strongly acidic; further simply “acidic”, and 6.5, slightly acidic to neutral; further simply “neutral”) to mimic gastrointestinal conditions (stomach and upper small intestine). To compare differences in dissolution profiles of IBU from solid CS-IBU lyophilizates at different pH values comprehensively, continuous (separate, individual) dissolution tests, one at pH 1.2 and another one at pH 6.5, were compared with the dissolution test performed in the whole interval of tested pH values discontinuously (1.2–6.5), i.e., discontinuous dissolution test. The hydrogels were prepared under different conditions (solvent and cosolvent composition), ensuring the creation of either a heterogeneous or homogeneous CS-IBU mixture. To evaluate the effect of CS itself on IBU solubility enhancement, we examined CS in its concentration cascade within all tested systems.

Lyophilizates were prepared from hydrogels containing CS with medium molecular weight (CS MMW) as a gelling agent at 3 individually chosen concentrations (0.2, 0.4, and 0.8% *w*/*w*) to assess the influence of CS concentration on IBU dissolution. Three systems of solvents were selected: (i) conventional acetic acid, where undissolved IBU forms suspension (named HAc system), (ii) a combination of ethanol with lactic acid (named EtOH system), or (iii) a combination of isopropyl alcohol with lactic acid (named IPA system) in which IBU and CS could coexist in the dissolved (homogeneous) form together. In the first step, the structure of lyophilizates was investigated under a scanning electron microscope (SEM) to evaluate the role of potential differences in the microstructures of CS-IBU lyophilizates, resulting from their homogeneous and heterogeneous hydrogels, on IBU dissolution. In the second step, the swelling index was determined to characterize the behavior of the CS-IBU lyophilizates in aqueous media with different pH values. In the final step, in vitro dissolution of IBU from the lyophilizates was designed to obtain a complex view on the role of particular parameters participating in the dissolution process. For each dissolution profile, kinetic data were calculated and a kinetic model was proposed.

## 3. Results and Discussion

### 3.1. SEM Analysis

**Figure 1:**
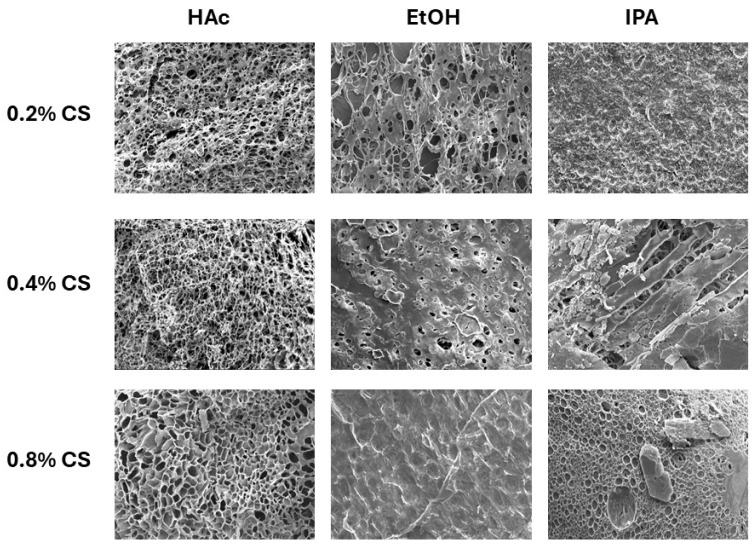
SEM micrographs of lyophilized CS-IBU hydrogels prepared using different solvents (HAc = acetic acid, EtOH = ethanol, IPA = isopropanol) and CS concentration (0.2%, 0.4%, 0.8%). Images were obtained at 500× magnification.

Microscopic images of the CS-IBU lyophilizates were made with a scanning electron microscope to evaluate the microstructure of the lyophilizates when the concentration of CS is increased, and in the case of solvent systems change. The illustrative images are depicted in Figure 1, where the measured systems, the CS-IBU lyophilizates, were prepared from corresponding hydrogels employing different solvents (HAc, EtOH, IPA) and CS concentrations (0.2%, 0.4%, 0.8%). The structure of the CS-IBU lyophilizates seems to be porous and sponge-like, more or less combined with compact glassy areas and/or lumps resembling IBU or CS crystals. Compact glassy areas (mainly on the surface) seem to prevail in homogeneous systems in the measured group of samples; however, the selection of measured points/areas within each sample is random; therefore, these observations cannot be considered completely invariant and definitive, and may vary from sample to sample.

The porosity study was performed to evaluate the SEM images. The results are shown in Table 2. Although porosity is slightly increasing with the amount of CS in the lyophilizates, the differences (trends) are small. The porosity of individual systems is similar and consistent within the measured CS-IBU lyophilizate systems. Summarizing, the SEM images were randomly selected to cover various parts of the lyophilizates; hence, they are illustrative. Anyway, the similarity of their porous microstructure was supported by consistent data from the porosity testing.

Although the microscopic structure of the lyophilizates affected IBU dissolution to some extent, this parameter may seem of less practical importance compared to IBU dissolution affected by CS concentration (see Section 2.9). An apparently greater influence of CS concentration on IBU dissolution was clearly demonstrated via the dissolution experiments and the found trends. Since the microscopic structure of the lyophilizate is considered a relatively highly variable parameter (influenced by multiple factors involved in both hydrogel preparation and hydrogel lyophilization as well), it can be more difficult to observe some significant trends in this context.

Nevertheless, the microscopic structure of the lyophilizates showed more visible impact on their swelling (see Section 2.8); therefore, it is necessary to take it into account in a complex evaluation of the behavior of CS-IBU lyophilizates in various acid-base systems mimicking GIT conditions.

### 3.2. FT−IR Analysis of CS-IBU Interactions

**Figure 2:**
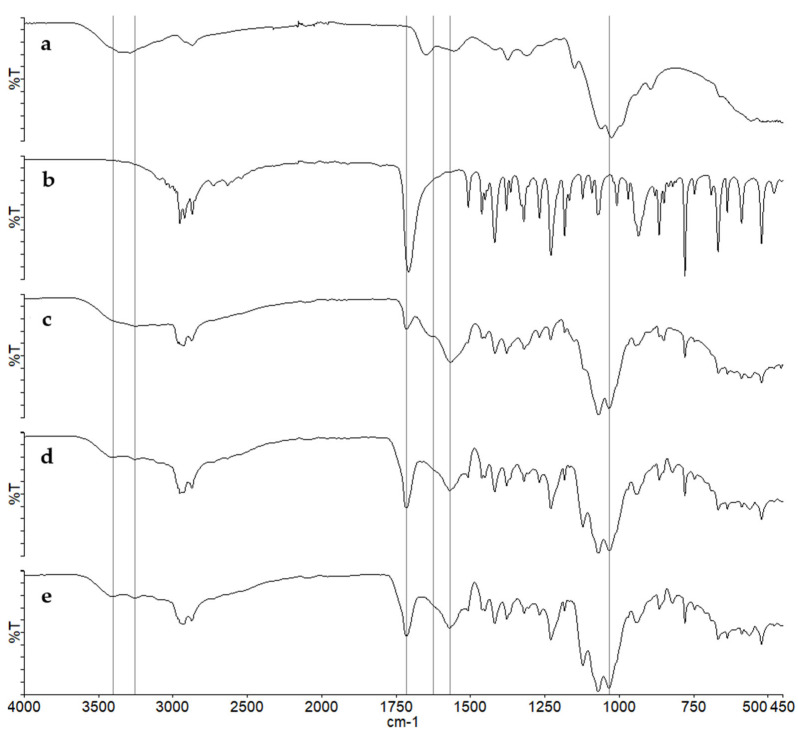
FT−IR spectra of (**a**) CS, (**b**) IBU, (**c**) 0.8% CS HAc, (**d**) 0.8% CS EtOH, and (**e**) 0.8% CS IPA samples. Gray vertical lines indicate the shifts in selected bands relative to native CS and IBU.

To confirm interactions between CS and IBU in lyophilized hydrogel samples, the FT−IR spectrum of each sample was acquired and compared with the spectra of native CS and IBU. For clarity, only the spectra of CS, IBU, and samples containing 0.8% CS are presented (Figure 2). The spectra of samples containing 0.2% and 0.4% CS exhibited highly similar bands to those of their 0.8% CS counterparts, differing only in the intensity of certain bands and insignificant shifts.

Native CS showed the characteristic FT−IR bands, including the broad O–H/N–H stretching region with maxima at 3356 and 3290 cm^−1^, the amide I band at 1652 cm^−1^, the amide II band at 1558 cm^−1^, glucosamine ring bands at 1151 and 895 cm^−1^, and C-O stretch of primary alcohol. Native IBU exhibited the typical spectral features, namely the C=O stretching band at 1708 cm^−1^, the C–O stretching band at 1230 cm^−1^, the split CH_3_ bend at 1379 and 1365 cm^−1^, the O–H deformation band at 935 cm^−1^, and the aromatic C-H bend at 779 cm^−1^.

In the lyophilized samples, bands characteristic of both CS and IBU remained detectable, although some weaker/overlapping bands (e.g., C-O-C asymmetric stretching bands in the EtOH and IPA samples) were more clearly resolved in the first-derivative spectra, thus confirming the presence of both components. However, compared with native CS, the amide I band shifted from 1652 cm^−1^ to 1636–1630 cm^−1^, the NH_2_ bending band shifted from 1558 cm^−1^ to 1567–1571 cm^−1^, and the peak maxima of the O–H/N–H stretching region were also shifted. Additionaly, the ibuprofen carbonyl band was shifted towards 1715 cm^−1^. The data is summarized in Table 3. These spectral changes suggest intermolecular interaction between CS and IBU, possibly via hydrogen bonding and/or other non-covalent interactions, most likely involving the ibuprofen carboxylic acid group and the amino/hydroxyl groups of chitosan. Similar interactions have also been reported previously for CS-IBU aerogels [23].

### 3.3. Swelling Index Determination

**Figure 3:**
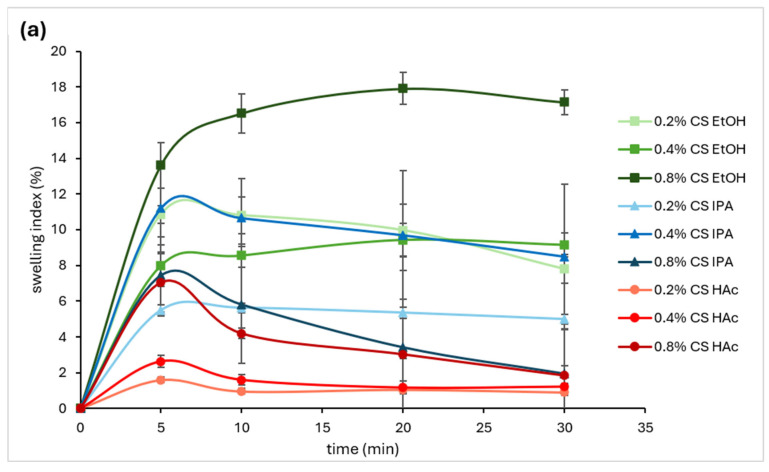

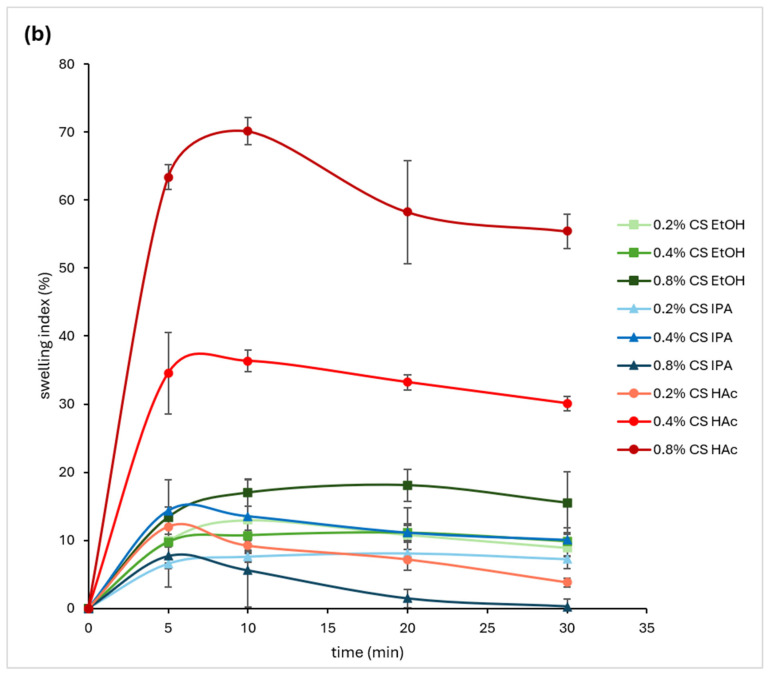
Swelling index of CS-IBU lyophilizates in different acid-base media. The concentration of CS was 0.2%, 0.4%, and 0.8%; the concentration of IBU was constant 0.4%. Lyophilizates prepared from homogeneous (IPA, EtOH) and heterogeneous (HAc) hydrogels were tested in the (**a**) acidic (pH 1.2) and (**b**) neutral (pH 6.5) media.

The swelling index (SI) was determined for each CS lyophilizate containing IBU in a medium with pH = 1.2 and 6.5 simulating the pH of gastric fluid and the environment of the beginning of the small intestine, respectively. The swelling ability of CS lyophilizates prepared from homogeneous (EtOH, IPA) and heterogeneous (HAc) hydrogels with different content of CS (0.2%, 0.4%, and 0.8%) was evaluated, and a graphical representation of the dependency of swelling index on time is illustrated in Figure 3.

The SI profiles indicated that the CS-IBU lyophilizates prepared from homogeneous (EtOH, IPA) and heterogeneous (HAc) hydrogels had mutually different swelling behavior. In the acidic environment (pH 1.2), HAc-derived lyophilizates (red profiles in Figure 3a) exhibited significantly lower SI values in comparison with IPA/EtOH-derived lyophilizates (blue/green profiles in Figure 3a). In the neutral environment (pH 6.5), HAc-derived lyophilizates (red profiles in Figure 3b) exhibited (for higher CS concentrations) significantly higher SI values in comparison with IPA/EtOH-derived lyophilizates (blue/green profiles in Figure 3b).

Multiple simultaneously acting factors can influence the SI profiles of the CS-IBU lyophilizates in different acid-base media. As one of them, these observations could be explained via different microscopic structures of the CS-IBU lyophilizates prepared from homogeneous and heterogeneous hydrogels. The prevailing glassier or porous surface structure of the CS-IBU lyophilizates prepared from homogeneous or heterogeneous hydrogels, respectively, logically explains a natural ability to incorporate a given solvent into the lyophilizate structure (a porous surface is supposed to undergo solvent penetration more easily than a glassy one), and, by that, swelling possibility.

When interpreting these observations, however, one should also take into consideration the potential loss of CS or IBU due to their intrinsic solubilities under given pH (IBU at pH 6.5, while CS at pH 1.2), which influences the calculation of a true SI value. Therefore, the combination of a more porous (more susceptible to erosion) structure and its simultaneous exposure to the environment supporting the solubility (and by that loss) of its building units (CS) seems to be a logical explanation of the lowest SI values found for the HAc-derived lyophilizates in the acidic environment (pH 1.2). Analogically, the combination of a more porous structure (favoring the solvent penetration) and its simultaneous exposure to the environment, suppressing the solubility (and by that preservation) of its building units (CS), seems to be a logical explanation of the highest SI values found for the HAc-derived lyophilizates in the neutral environment (pH 6.5). Thinking in the same way, a less porous and glassier (less susceptible to erosion) surface structure, despite its exposure to the environment supporting the solubility of its building units (CS), seems to be a logical explanation of the highest SI found for the IPA/EtOH-derived lyophilizates in the acidic environment (pH 1.2). Analogically, a less porous and glassier surface structure (less favorable to the solvent penetration), even though exposed to the environment, preserving its building units (CS), seems to be a logical explanation of the lowest SI found for the IPA/EtOH-derived lyophilizates in the neutral environment (pH 6.5).

It is clear that in addition to the factors mentioned above in the text, other factors, such as simultaneous IBU dissolution, variable topography of the lyophilizate surfaces, etc., are among those affecting the SI profiles too. It is understandable that, given the complexity of factors affecting the SI values calculated for the CS-IBU lyophilizates, subtle differences in the swelling behavior of different CS-IBU lyophilizates cannot be recognized and described/explained unambiguously; however, the main trends, as mentioned above, can be valuable insights for the overall understanding of the action of CS-IBU lyophilizates in various acid-base media mimicking GIT conditions.

### 3.4. In Vitro Drug Release and Dissolution

**Figure 4:**
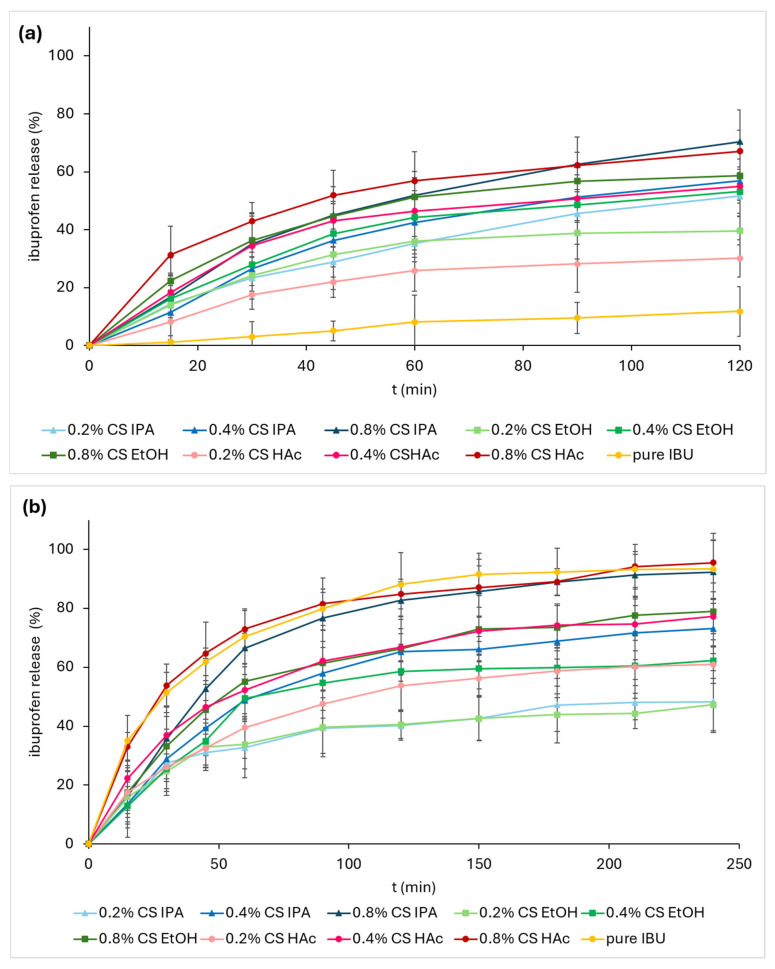
Dissolution profiles of IBU from CS-IBU lyophilizates at different acid-base conditions in two separate continuous dissolution systems: (**a**) pH 1.2, (**b**) pH 6.5. The yellow profile refers to IBU dissolution in a CS-free system.

**Figure 5:**
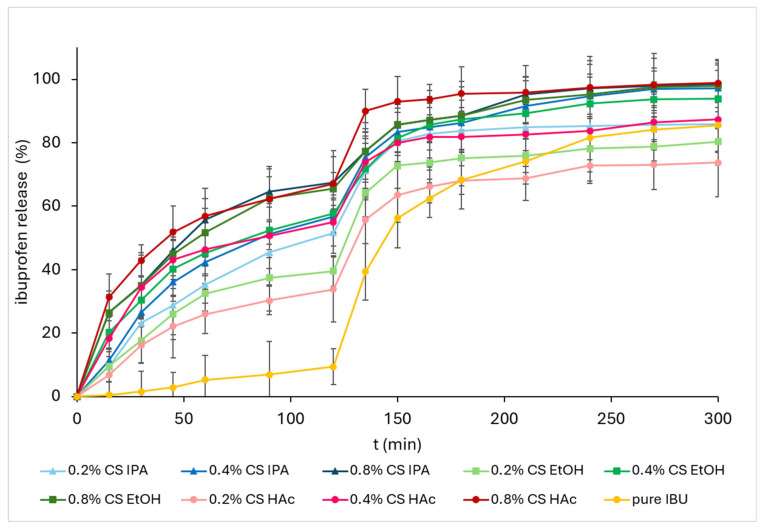
Dissolution profiles of IBU from CS-IBU lyophilizates at different acid-base conditions in one discontinuous dissolution system mimicking GIT conditions. The dissolution system started with a pH of 1.2 and continued for 120 min, then with a pH of 6.5 for 300 min. The yellow profile refers to IBU dissolution in a CS-free system.

Drug release (from the solid matrix) and dissolution (in given media), simply a dissolution test, by in vitro diffusion of IBU from CS lyophilizates were evaluated using a paddle apparatus defined in the European Pharmacopeia 11th edition. The dissolution test was designed in a way (i) to compare the influence of pH on IBU release/dissolution from CS-IBU lyophilizates in two separate continuous acid-base media relevant to individual parts of the GIT (Figure 4), and (ii) to simulate the passage of medicine through the GIT in one discontinuous system with a variable pH profile (Figure 5).

In the first case, the dissolution was carried out at pH values of either 1.2 (Figure 4a) or 6.5 (Figure 4b), with an approximately constant pH profile over the entire time span (from 0 to 120 or 250 min). A high molar excess of hydrochloric acid over CS glucosamine preserves an acidic environment despite extensive CS protonation, whereas in a near-neutral environment, low CS protonation/solubility (CS pKa~6.3) does not significantly affect the medium’s pH. In the second case, the dissolution started in a medium with a pH of 1.2, and after 120 min, the pH was rapidly adjusted (within 5 min) to pH 6.5, simulating the passage of the drug from the gastric to duodenal medium, and the dissolution test continued for another 180 min. The resulting dissolution profiles are illustrated in Figure 5. The amounts of released/dissolved IBU and the rate of IBU dissolution were compared within the group of CS lyophilizates with different homogeneity and content of CS on one side (i.e., parameters of the lyophilizates) and for different pH values of the dissolution media on the other side (i.e., parameters of the media). The IBU dissolution was also compared with respect to the pH profile of the dissolution system, i.e., constant vs. variable pH profile.

In this comparative study, the CS-IBU lyophilizates prepared in different ways with respect to concentrations of CS (0.2, 0.4, and 0.8% *w*/*v*) and solvent/cosolvent composition of the gel mixture ((i) acetic acid; (ii) lactic acid and ethanol; and (iii) lactic acid and isopropyl alcohol) showed different impacts of their particular parameters for IBU dissolution. It can be seen that there is no significant trend of hydrogel preparation and homogeneity on the dissolution of IBU from the CS-IBU lyophilizates at the medium and high CS concentrations and acidic pH; see the dissolution profiles for 0.4 and 0.8% CS depicted in Figure 4a. On the other hand, the trend is more pronounced at low CS concentrations, where the heterogenous CS-IBU system showed significantly lower IBU dissolution than each of the homogeneous ones (Figure 4a, 0.2% CS). In general, however, the influence of the microstructure of the CS-IBU lyophilizate on the dissolution of IBU in acidic media appears to be of minor practical importance. Compared to the microstructure effect, generally a significant trend can be seen for the CS concentrations in the hydrogel, demonstrated by increased IBU dissolution from CS-IBU lyophilizates with higher CS concentrations in the acidic dissolution system (a 1.3–2.2-fold IBU solubility enhancement within 120 min when comparing the 0.2% and 0.8% CS-IBU systems). Hence, CS served as an efficient IBU solubilizer in an acidic environment, providing ca. 15–28-fold IBU solubility enhancement even within 15 min when comparing the free IBU system (i.e., without any CS presence) and the 0.8% CS-IBU systems. The situation is quite different in an almost neutral dissolution system (pH 6.5); see Figure 4b. Here, an intrinsic IBU solubility (IBU pKa~5.2) is considerably higher than it is at acidic pH (1.2); therefore, IBU can also be dissolved without any CS intervention. In this dissolution system, a solid CS-IBU lyophilizate structure (responsible for IBU entrapment) is eroding/dissolving continually and relatively slowly, resulting in a prolonged IBU release (compare free and CS-IBU dissolution profiles in Figure 4a,b). When investigating the influence of the microstructure of the CS-IBU lyophilizates on the dissolution of IBU in the neutral medium, the CS-IBU lyophilizates prepared from the heterogeneous hydrogel systems (HAc) provided an increased IBU dissolution compared to the CS-IBU lyophilizates prepared from the homogeneous hydrogel systems (EtOH, IPA). It may be due to the prevailing porous sponge-like structure (more easily eroding) in the first case and probably a higher ratio of glassy structure (more difficult to erode) in the second case (mainly observed in the EtOH system). It may also depend on the ratio of CS-entrapped and free (excluded) IBU in homogeneous and heterogeneous systems and on other material-related factors. Surprisingly, the dissolution of IBU within the group of the tested CS-IBU systems increased with the elevated CS concentration (e.g., a 1.6–2.1-fold IBU solubility enhancement within 120 min when comparing 0.2% and 0.8% CS-IBU systems) also in the neutral medium. A possible explanation is that CS can act as an effective solubilizer for IBU in both acidic and neutral acid-base conditions as well. In an acidic environment, CS-supported IBU solubilization is supposed to be due to IBU association with CS predominantly through hydrogen bonding and hydrophobic interactions while in a neutral environment, besides those interactions, ionic pairing between protonated amines on chitosan and the carboxylate of IBU (where IBU can serve as a proton donor and CS as an effective proton acceptor) may be an additional interaction of a higher significance that modifies (additionally amplifies) IBU solubility. Thus, in the neutral environment, the competition between intrinsic IBU solubility and CS-amplified IBU solubilization and CS lyophilizate solid matrix-driven IBU trapping is supposed to be responsible for the overall IBU dissolution effect. Based on the above observations and discussion, in neutral environments (mimicking the upper small intestine), CS-IBU lyophilizates prepared from homogeneous hydrogels and, atypically, lower CS concentrations in CS-IBU lyophilizates could be among the effective tools for prolonging IBU release (apparently, related to the given CS/IBU concentration ratio). As an additional finding, CS-IBU lyophilizates (mainly those prepared from heterogeneous hydrogels) possessing higher CS concentrations can provide (in neutral environments) IBU dissolution comparable to pure (i.e., CS matrix-free) IBU dissolution. Thus, matrix-free and proper CS matrix-assisted IBU systems can represent two alternatives for introducing an equivalent amount of dissolved IBU into neutral environments.

When comparing data from the dissolution experiments conducted in the individual constant pH systems (Figure 4) with the discontinuous variable pH system (Figure 5), IBU dissolution profiles generally match each other in acidic environments of both systems, while they differ from each other in several aspects in neutral environments of both systems. The first observation is logical, because the experiments were carried out under the same conditions. In the second case, however, the starting conditions, influencing the whole dissolution process, were quite different. The measurement at pH 6.5 started with the solid CS-IBU lyophilizate in the continuous system (Figure 4b), but it started with already pre-dissolved CS-IBU lyophilizate in the discontinuous system (Figure 5, after pH change, i.e., from 120 min). Therefore, differences among the IBU dissolution profiles can be attributed mainly to a different degree/impact of the CS lyophilizate solid matrix-driven IBU trapping and the CS-IBU lyophilizate microstructure character, i.e., material-related factors in the neutral media of the continuous and discontinuous dissolution systems (compare, e.g., 0.2% CS-assisted HAc vs. EtOH/IPA dissolution profiles of both systems). The effect of CS concentration on the IBU dissolution in neutral media, however, is similar in both continuous and discontinuous dissolution systems; i.e., the dissolved amount of IBU is enhanced with higher CS concentration, while the dissolution rate is prolonged with lower CS concentration (also towards the free IBU system). An enhanced IBU dissolution in the CS matrix-assisted dissolution system under both acidic and neutral conditions (mimicking those of GIT), with an additional possibility to modulate/prolong IBU release (mainly under neutral conditions), may represent one of the most important outputs of practical significance.

### 3.5. Determination of Drug Release Kinetics

Drug release kinetics were compared through the coefficient of determination (R2) expressed for the regression line of five kinetic models (zero order, first order, Higuchi, Korsmeyer–Peppas, and Hixson–Crowell); see data in Appendix A, Table A1.

In Table 4, there are kinetic constants for all kinetic models, and Table 5 shows the mean dissolution time (MDT) calculated for CS-IBU lyophilizates in each dissolution system (pH 1.2, pH 6.5, discontinuous pH 1.2–6.5).

The values obtained from in vitro dissolution studies in acidic media (pH 1.2) indicate that the ibuprofen release from chitosan-based lyophilizates follows mostly the Higuchi model. This kinetic drug release model commonly describes the dissolution of a low-solubility drug (here, IBU in a strongly acidic environment), enhanced by a solubility enhancer (in our case, dissolved CS) in a solid matrix system; thus, it may be well applied to diffusion-controlled systems where the enhancer increases the drug’s solubility in the medium. The fact that the dissolution kinetics in most cases correspond to the Higuchi kinetic model indicates that the CS lyophilizates bind ibuprofen and, through (rapid) gradual erosion of the CS polymer matrix, the drug (either free or, mainly, as a CS associate) diffuses into the environment. Some (random) deviations from this model (towards the Korsmeyer–Peppas kinetic model) may be due to measurement errors (see very close values of the determination coefficients of these two models in these cases). Nevertheless, even the Korsmeyer–Peppas kinetic model (describing drug release kinetics through multi-phase processes of diffusion and erosion within the polymer matrix) would not exclude the above-mentioned facts related to the dissolution of IBU in the CS matrix-assisted acidic dissolution system.

Somewhat similar results were obtained in neutral media (pH 6.5). The Higuchi kinetic model also well-describes drug release from a solid matrix system as a diffusion process. It is primarily applied to solid dispersions, transdermal systems, and matrix tablets, indicating a release rate that decreases over time. Here, the Higuchi kinetic model (indicating slow, gradual erosion of the polymer matrix and subsequent drug diffusion into the environment) prevails again. Some (random) deviations from this model (towards the Korsmeyer–Peppas kinetic model) can be explained analogically as in the previous paragraph. Some other indications of a possible first-order kinetic model may suggest the different importance of individual dissolution mechanisms participating in acidic and neutral environments. A first-order kinetic model describes a process in which the release rate of a drug is proportional to the remaining amount of solid drug and is often used for water-soluble drugs in porous matrices. It indicates that the amount of drug released decreases over time as the concentration gradient declines. This kinetic model was indicated here for most 0.8% CS lyophilizate systems, which could be explained via CS-amplified IBU dissolution and/or major distribution of solid IBU out of the CS lyophilizate solid structure (i.e., dissolution of excluded/free IBU).

As expected, the IBU dissolution in the acidic region of the first (continuous 0–120 min) part of the discontinuous (0–300 min) dissolution system followed mostly the Higuchi kinetic model (comparable with the individual continuous acidic system). Some (random) deviations from this model (one and one towards the Korsmeyer–Peppas and the first-order kinetic model) may be due to measurement errors or the models’ similarity for the described systems (see very close values of the determination coefficients of these models in these cases). On the other hand, kinetic models for the discontinuous and continuous neutral dissolution systems differ from each other to a greater extent. A higher impact of the solid CS matrix on the IBU entrapment, combined with good intrinsic IBU solubility in the continuous system, resulted in IBU release following mainly the Higuchi kinetic model and possibly the first-order kinetic model. However, in the discontinuous dissolution system with an already (within 0–120 min) pre-dissolved CS matrix (resulting in a suppressed impact of the solid CS matrix on IBU entrapment), the first-order kinetic model was more relevant and best fit all these dissolution profiles. Hence, in the neutral medium of the discontinuous dissolution systems (135–300 min), a higher degree of the free (originally free plus previously released) drug, exhibited to the environment in which it can well dissolve, may be expected. At the same time, however, the CS matrix (with a temporally varying solid/liquid CS ratio) can still affect the dissolution of IBU.

The relatively high Higuchi kinetic constants (see Table 4) suggest that drug release from the lyophilized CS-IBU systems was predominantly diffusion-controlled. In addition, increased Hixson–Crowell constants observed for IPA-based formulations indicate that structural changes and matrix erosion also contributed to the release process. The obtained kinetic behavior correlated well with the porous morphology observed by SEM analysis.

The MDT values are shown in Table 5. Generally, higher MDT values indicated a more prolonged and controlled drug release. All formulations showed increased MDT values at neutral pH compared with acidic pH and after the pH change, confirming the pH-responsive behavior of the lyophilized hydrogels and their ability to sustain drug release under gastrointestinal conditions. Possible fluctuations and any trends in MDT values across the dissolution systems can be caused by the competition of CS-IBU associate-enhanced dissolution, CS solid matrix suppressed dissolution, and intrinsic IBU dissolution under different acid-base conditions.

Linearity between MDT and concentration of CS was observed only at HAc systems (correlation coefficients were in the range of 0.8392–0.9895). The absence of a clear linear relationship in IPA and EtOH systems indicates that MDT was influenced not only by CS concentration but also by structural and morphological factors affecting drug diffusion and matrix swelling.

## 4. Conclusions

CS-IBU hydrogel lyophilizates were demonstrated as promising stimuli (pH)-responsive carrier systems for a model NSAID, namely IBU, in model dissolution systems mimicking continuous or discontinuous GIT conditions. Despite the complexity of factors affecting the behavior of the CS-IBU lyophilizates, several significant trends in their action and parameters responsible for their action under different acid-base conditions could be well distinguished, based on the experiments performed.

The intrinsic IBU dissolution was affected significantly by a CS concentration effect and a CS solid matrix effect within each tested CS-IBU lyophilizate subgroup (HAc, IPA, EtOH subgroups differing by their preparation and apparently also in their microstructures), with different impact ratio of these effects under different acid-base conditions (well-soluble CS in a acidic environment offered association with IBU in the CS liquid phase, while less soluble CS in a neutral environment offered capture of IBU in the CS solid phase). It indicated pH-dependent distribution of IBU into four phases: liquid CS phase (supported IBU solubility as CS-IBU associate, responsible for IBU solubility enhancement), solvent phase (natural IBU solubility in given medium, non-associated dissolved IBU), solid CS phase (IBU trapped in solid CS matrix, responsible for IBU prolonged release), and solid IBU phase (insoluble IBU portion in given medium, non-associated excluded IBU). The release from the solid CS matrix and dissolution of a low-solubility drug (IBU) enhanced by a solubility enhancer (dissolved CS) was confirmed by the Higuchi kinetic model for acidic as well as neutral media of the continuous dissolution systems, while the drug release in neutral pH of the discontinuous dissolution system with already pre-dissolved CS matrix was supported by the first-order kinetic model. An effect of CS-IBU lyophilizate microstructure (indicated by scanning electron microscope), accompanying and additionally modifying these distribution equilibria, was distinguished (to some measure) by dissolution and swelling index profiles. More porous or glassier microstructures for respective heterogeneous Hac- or homogeneous EtOH/IPA-based systems allowed varying degrees of solvent penetration into the structure of the solid lyophilizate, and, by that, its easier swelling (in neutral pH) or decay (in acidic pH). Thus, compared to the CS matrix-free IBU dissolution systems, the competition between supporting and suppressing CS-mediated IBU dissolution effects can be effectively controlled and practically utilized for an IBU solubility enhancement in the acidic environment and a prolonged IBU release of different degrees in the neutral environment.

These results indicated the following practical options for rationally designed CS-IBU lyophilized hydrogel formulations intended for oral administration (considered as a model example):

(i) CS-induced IBU dissolution takes effect in the gastric part of the GIT, where the concentration of dissolved IBU can be controlled/increased by the CS concentration; a CS-induced IBU solubility enhancement provides benefits of higher and earlier gastric drug absorption, allowing a dose reduction (or an enhancement of the therapeutic effect at the same dose) and an earlier onset of drug effect;
(ii) Pre-dissolved CS-IBU lyophilizate enables, depending on the given CS/IBU ratio, duodenal absorption of the drug after rapid (at a higher CS/IBU ratio) or prolonged (at a lower CS/IBU ratio) dissolution of the drug;
(iii) When inserting solid CS-IBU lyophilizate (e.g., protected against the gastric conditions by proper coating) into the duodenum, the drug release and dissolution could be controlled by lyophilizate microstructure (glassier surfaces resulting from homogeneous hydrogel precursors, less prone to erosion, prolong IBU release and vice versa for more porous heterogeneous systems) and/or CS/IBU ratio (a lower CS/IBU ratio prolongs IBU release and vice versa for a higher CS/IBU ratio);
(iv) The bioavailability of free IBU and CS-associated IBU may differ from each other, which can be utilized for additional optimization of the IBU action in biological systems (not studied in this work on a real biological system, but worthy of future study);
(v) CS protective effects (largely due to its high mucoadhesivity, biocompatibility, and its intrinsic antimicrobial, anti-inflammatory, and wound-healing properties) can be considered as additional benefits when using CS in combination with IBU or, analogically, other NSAIDs.

## Figures and Tables

**Table 1:** Equations used to calculate kinetic models.

**Kinetic Model**	Equation	Parameters
zero-order kinetics	Q_t_ = Q_0_ + kt	Q_t_ = amount of drug released in time t Q_o_ = initial amount of drug k = zero-order rate constant t = time
first-order kinetics	ln(Q_0_ − Q_t_) = lnQ_0_ − kt	Q_0_ = initial amount of drug Q_t_ = amount of drug released in time t Q_0_ − Q_t_ = amount of drug remaining in the dosage form k = first-order rate constant
Higuchi	Q_t_ = k_H_t^1/2^	Q_t_ = cumulative amount of released drug k_H_ = Higuchi rate constant t^1/2^ = square root of time
Korsmeyer–Peppas	$\frac{M_{t}}{M_{\infty }}=kt^{n}$	$M_{t}/M_{\infty }$ = fraction of released drug at time t k = rate constant n = release exponent
Hixson–Crowell	Q_0_^1/3^ − Q_t_^1/3^ = kt	Q_0_ = initial amount of drug Q_t_ = amount of drug remaining in the system at time t k = Hixson–Crowell rate constant

**Table 2:** Porosity of CS lyophilizates.

Sample	Measurement No.			Porosity (%) Average	±SD (%)
	1	2	3		
HAc					
0.2% CS	39.34	43.75	42.10	41.73	1.8
0.4% CS	45.29	41.60	40.77	42.56	2.0
0.8% CS	40.37	44.23	44.46	43.02	1.8
IPA					
0.2% CS	46.45	42.82	39.03	42.77	3.0
0.4% CS	47.93	36.70	45.64	43.42	4.8
0.8% CS	46.6	48.08	45.46	46.71	1.1
EtOH					
0.2% CS	38.28	45.85	36.66	40.26	4.0
0.4% CS	39.51	44.75	41.03	41.76	2.2
0.8% CS	46.55	48.07	40.36	44.99	3.3

**Table 3:** Assignment of characteristic FT−IR bands of CS, IBU, and 0.8% CS samples. Significant shifts in band positions relative to native CS and IBU are highlighted in bold and correspond to the gray lines in Figure 2.

Vibration	Wavenumber (cm^−1^)				
	CS	IBU	0.8% CS HAc	0.8% CS EtOH	0.8% CS IPA
O-H/N-H stretch	**3356; 3290**		**3413; 3250**	**3408; 3256**	**3408; 3256**
C-H stretch	2872; 2920	2954; 2922; 2869	2966; 2928; 2872	2951; 2929; 2872	2951; 2929; 2872
C=O stretch (carboxyl)		**1708**	**1715**	**1715**	**1715**
CH_3_ symmetric bend	1375	1379; 1365	1378; 1366	1378; 1366	1378; 1365
C=O (Amide I)	**1652**		**1636**	1630 *	1630 *
N-H bend (Amide II)	**1558**		**1567**	**1570**	**1571**
C-O stretch (carboxyl)		1230	1230	1230	1230
O-H bend	943	935	940	940	940
C-O-C asymmetric stretch (saccharide)	1151		1153	1155 *	1155 *
C-O stretch (CH_2_-OH)	**1026**		**1034**	**1033**	**1034**
C-O-C symmetric stretch (saccharide)	895		897	897	897
aromatic C-H bend		779	779	779	779

* Weak band shoulder.

**Table 4:** Drug release kinetic constant k calculated from data corresponding to the dissolution profiles of CS-IBU lyophilizates obtained in different dissolution systems (pH 1.2, pH 6.5, discontinuous pH 1.2–6.5).

	Kinetic Constants (k)				
Lyophilizates	Zero Order	First Order	Higuchi	Korsmeyer–Peppas	Hixson–Crowell
pH 1.2					
0.2% CS in HAc	14.78	0.18	23.58	0.04	0.26
0.2% CS in IPA	25.26	0.37	38.30	0.03	0.50
0.2% CS in EtOH	18.99	0.25	31.08	0.03	0.35
0.4% CS in HAc	25.74	0.39	41.93	0.02	0.52
0.4% CS in IPA	28.94	0.44	44.24	0.03	0.59
0.4% CS in EtOH	25.76	0.39	40.89	0.02	0.52
0.8% CS in HAc	29.52	0.53	49.06	0.02	0.66
0.8% CS in IPA	34.82	0.62	53.65	0.02	0.79
0.8% CS in EtOH	27.49	0.44	45.00	0.02	0.58
pH 6.5					
0.2% CS in HAc	13.22	0.23	31.71	0.03	0.29
0.2% CS in IPA	9.72	0.14	23.82	0.03	0.19
0.2% CS in EtOH	8.85	0.14	22.13	0.03	0.17
0.4% CS in HAc	15.97	0.35	38.91	0.02	0.41
0.4% CS in IPA	16.32	0.14	39.13	0.03	0.87
0.4% CS in EtOH	13.49	0.23	33.17	0.03	0.29
0.8% CS in HAc	17.97	0.69	45.71	0.02	0.65
0.8% CS in IPA	20.66	0.64	49.94	0.02	0.66
0.8% CS in EtOH	17.00	0.37	40.96	0.02	0.89
Region (pH 1.2) before the pH change					
0.2% CS in HAc	16.61	0.21	25.83	0.04	0.30
0.2% CS in IPA	25.53	0.37	38.74	0.03	0.50
0.2% CS in EtOH	19.78	0.25	30.84	0.04	0.36
0.4% CS in HAc	24.84	0.37	40.92	0.02	0.50
0.4% CS in IPA	27.98	0.41	43.25	0.03	0.57
0.4% CS in EtOH	26.38	0.41	42.25	0.02	0.54
0.8% CS in HAc	28.48	0.51	47.85	0.02	0.64
0.8% CS in IPA	31.17	0.55	50.10	0.02	0.70
0.8% CS in EtOH	29.87	0.53	48.05	0.02	0.66
Region (pH 6.5) after the pH change					
0.2% CS in HAc	5.42	0.16	20.71	0.02	0.18
0.2% CS in IPA	3.65	0.18	14.15	0.01	0.17
0.2% CS in EtOH	4.34	0.16	16.56	0.02	0.17
0.4% CS in HAc	3.76	0.21	14.25	0.01	0.18
0.4% CS in IPA	7.30	0.81	27.68	0.02	0.55
0.4% CS in EtOH	6.58	0.51	25.19	0.02	0.40
0.8% CS in HAc	2.82	0.71	10.70	0.01	0.37
0.8% CS in IPA	7.78	1.01	26.64	0.02	0.62
0.8% CS in EtOH	6.62	0.88	25.17	0.02	0.55

**Table 5:** Mean dissolution time (MDT) calculated for dissolution profiles of CS-IBU lyophilizates in different dissolution systems (pH 1.2, pH 6.5, discontinuous pH 1.2–6.5).

MDT (h)	System								
	0.2% CS HAc	0.4% CS HAc	0.8% CS HAc	0.2% CS IPA	0.4% CS IPA	0.8% CS IPA	0.2% CS EtOH	0.4% CS EtOH	0.8% CS EtOH
pH 1.2	0.550	0.522	0.484	0.699	0.669	0.660	0.471	0.575	0.486
pH 6.5	0.926	0.889	0.782	0.878	0.965	0.895	0.865	0.802	0.955
pH change	1.655	1.275	1.109	1.391	1.499	1.235	1.557	1.366	1.273

## Data Availability

The original contributions presented in this study are included in the article. Further inquiries can be directed to the corresponding author.

## Appendix A

**Table A1:** Drug release kinetics calculated from data corresponding to the dissolution profiles of CS-IBU lyophilizates obtained in different dissolution systems (pH 1.2, pH 6.5, discontinuous pH 1.2–6.5).

	Coefficient of Determination (R^2^) *				
Lyophilizates	Zero Order	First Order	Higuchi	Korsmeyer–Peppas	Hixson–Crowell
pH 1.2					
0.2% CS in HAc	0.8114	0.8409	0.9558	0.9065	0.8313
0.2% CS in IPA	0.9235	0.9644	0.9815	0.9888	0.9533
0.2% CS in EtOH	0.7634	0.8034	0.9458	0.9103	0.7906
0.4% CS in HAc	0.7796	0.8541	0.9566	0.9072	0.8302
0.4% CS in IPA	0.8919	0.9461	0.9636	0.9403	0.9302
0.4% CS in EtOH	0.8326	0.8941	0.9698	0.9461	0.8749
0.8% CS in HAc	0.7534	0.8755	0.9625	0.9748	0.8375
0.8% CS in IPA	0.8898	0.9633	0.9766	0.9525	0.9481
0.8% CS in EtOH	0.7765	0.8544	0.9605	0.9386	0.8303
pH 6.5					
0.2% CS in HAc	0.8139	0.8946	0.9656	0.9699	0.8704
0.2% CS in IPA	0.7520	0.8281	0.9313	0.8853	0.8038
0.2% CS in EtOH	0.7035	0.7887	0.9079	0.9132	0.7521
0.4% CS in HAc	0.7746	0.9088	0.9488	0.9452	0.8700
0.4% CS in IPA	0.7993	0.9054	0.9475	0.9090	0.8735
0.4% CS in EtOH	0.7178	0.7754	0.8957	0.8685	0.7661
0.8% CS in HAc	0.6914	0.9578	0.9016	0.9086	0.8931
0.8% CS in IPA	0.7693	0.9580	0.9278	0.8803	0.9056
0.8% CS in EtOH	0.7922	0.9268	0.9497	0.9164	0.8879
Region (pH 1.2) before the pH change					
0.2% CS in HAc	0.8922	0.9230	0.9683	0.9291	0.9132
0.2% CS in IPA	0.9405	0.9797	0.9724	0.9547	0.9689
0.2% CS in EtOH	0.8848	0.9174	0.9655	0.9538	0.9072
0.4% CS in HAc	0.7844	0.8598	0.9560	0.9026	0.8356
0.4% CS in IPA	0.9061	0.9614	0.9720	0.9411	0.9453
0.4% CS in EtOH	0.8594	0.9353	0.9900	0.9805	0.9126
0.8% CS in HAc	0.7586	0.8826	0.9614	0.9756	0.8442
0.8% CS in IPA	0.8494	0.9395	0.9851	0.9788	0.9144
0.8% CS in EtOH	0.8523	0.9436	0.9900	0.9888	0.9182
Region (pH 6.5) after the pH change					
0.2% CS in HAc	0.7841	0.8421	0.8191	0.8217	0.8239
0.2% CS in IPA	0.5426	0.6300	0.5848	0.6067	0.6004
0.2% CS in EtOH	0.7408	0.8211	0.7725	0.7744	0.7954
0.4% CS in HAc	0.8003	0.8752	0.8226	0.8257	0.8534
0.4% CS in IPA	0.8958	0.9835	0.9223	0.9276	0.9715
0.4% CS in EtOH	0.7588	0.9168	0.7966	0.8015	0.8730
0.8% CS in HAc	0.8826	0.9834	0.9089	0.9250	0.9689
0.8% CS in IPA	0.8160	0.9775	0.8771	0.8890	0.9492
0.8% CS in EtOH	0.8637	0.9849	0.8932	0.8990	0.9678

* The best (highest) values of the coefficients of determination are highlighted in gray cells.
